# Multiple evolutionary origins of giant viruses

**DOI:** 10.12688/f1000research.16248.1

**Published:** 2018-11-22

**Authors:** Eugene V. Koonin, Natalya Yutin

**Affiliations:** 1 National Center for Biotechnology Information, National Library of Medicine, National Institutes of Health, Bethesda, MD, USA

**Keywords:** giant viruses, virus evolution, virus-host interaction, gene gain; gene loss, phagocytosis

## Abstract

The nucleocytoplasmic large DNA viruses (NCLDVs) are a monophyletic group of diverse eukaryotic viruses that reproduce primarily in the cytoplasm of the infected cells and include the largest viruses currently known: the giant mimiviruses, pandoraviruses, and pithoviruses. With virions measuring up to 1.5 μm and genomes of up to 2.5 Mb, the giant viruses break the now-outdated definition of a virus and extend deep into the genome size range typical of bacteria and archaea. Additionally, giant viruses encode multiple proteins that are universal among cellular life forms, particularly components of the translation system, the signature cellular molecular machinery. These findings triggered hypotheses on the origin of giant viruses from cells, likely of an extinct fourth domain of cellular life, via reductive evolution. However, phylogenomic analyses reveal a different picture, namely multiple origins of giant viruses from smaller NCLDVs via acquisition of multiple genes from the eukaryotic hosts and bacteria, along with gene duplication. Thus, with regard to their origin, the giant viruses do not appear to qualitatively differ from the rest of the virosphere. However, the evolutionary forces that led to the emergence of virus gigantism remain enigmatic.

## Introduction

Viruses are obligate intracellular parasites that are generally considered to be tiny compared with cellular life forms. The classic operational definition of a virus, going back to the seminal experiments of Iwanowski and Beijerinck in the late 19th century, is that of a “filterable infectious agent” (that is, one that passes through 0.3-μm “sterilizing” filters) ^1, 2^. However, this definition was shattered by the 2003 discovery of the mimivirus (after “mimicking microbes”) that had typical icosahedral virions that, however, at 0.7 μM in diameter exceeded in size the smallest bacteria and archaea and accordingly were retained by sterilizing filters ^3^. Obviously, owing to their size, the giant viruses have been routinely missed in previous experiments. The genome of the mimivirus is commensurately huge by the standards of the virosphere and, at 1.1 Mb, is larger than the genomes of many parasitic bacteria and roughly the same size as the smallest genomes of free-living prokaryotes ^4^. The gene composition of the mimivirus presented a mix between genes shared with other viruses, those not typically found in other viruses but universal in cellular life forms, and ORFans, genes without detectable homologs. The characteristic virus genes of the mimiviruses placed it confidently within the nucleo-cytoplasmic large DNA viruses (NCLDVs), an expansive group of double-stranded DNA (dsDNA) viruses that reproduce mostly in the cytoplasm of diverse eukaryotes. As originally defined, the NCLDVs included the families *Poxviridae*, *Asfarviridae*, *Iridoviridae*, *Ascoviridae*, and *Phycodnaviridae*. Phylogenomic analysis has shown that, with some exceptions, these viruses share a core set of about 40 genes that encode key proteins required for virion structure formation and replication, indicating monophyletic origin of the entire group from a common virus ancestor. The mimivirus was found to encompass nearly all core NCLDV genes and preferentially clustered with phycodnaviruses in the phylogenetic trees of the core genes. Thus, the evolutionary analysis of the core genes suggested that the mimivirus was a typical NCLDV, even if “overgrown”. However, the universal cellular genes—in particular, those for translation system components, such as aminoacyl-tRNA synthetases (aaRSs)—seemed to point to a different history. In the respective phylogenetic trees, the mimivirus did not belong within any of the three domains of cellular life (bacteria, archaea, or eukaryotes) but rather formed a distinct branch. These observations have triggered the “fourth domain hypothesis”, according to which giant viruses evolved from cellular ancestors, most likely of an extinct fourth domain, via the reductive evolution route ^5– 8^.

The fourth domain scenario remained unclear on how to reconcile the presence of the complete genetic core of the NCLDVs and the genes for universal cellular genes in the giant virus genome. Nevertheless, for the next decade, the debate on giant viruses largely revolved around this hypothesis. The idea of the fourth domain has been strongly promoted ^9– 13^ but also vigorously contested, both on technical and on more general biological grounds ^14– 18^. In particular, shortly after the discovery of the mimivirus, it was shown by phylogenetic analysis that different genes in this virus appear to have different origins (eukaryotic, bacterial, or viral), leading to the notion of “giant viruses [as] giant chimeras” and to the more general conclusion that viruses are “gene robbers” par excellence ^14, 19– 23^. This discussion has even extended to a fascinating debate on the fundamental nature of viruses (giant but not only), whether they should be considered “alive” or not and whether or not viruses could, in principle, belong in the tree of life ^21, 24, 25^.

The seminal discovery of the mimivirus that has changed the very concept of a virus has strongly stimulated the interest of virologists in the NCLDVs. During the next decade, numerous relatives of the mimivirus as well as several additional groups of the NCLDVs, including new giant viruses, have been identified, primarily by co-cultivation with *Acanthamoeba*, or in some cases other amoebas, but also by isolation of viruses from marine protists and by methods of metagenomics ^26– 31^. The size records set by the mimivirus have been eclipsed by pandoraviruses that have virions of about 1 by 0.5 µm and genomes of more than 2.5 Mb ^32, 33^ and pithoviruses that currently hold the record of virion size (about 1.5 by 0.5 µm), albeit with considerably smaller genomes ^34^. The virions of both pandoraviruses and pithoviruses have unique, asymmetrical, amphora-like shapes that do not at all resemble the typical icosahedral virions of most of the NCLDVs (including the mimiviruses). *Mollivirus sibericum*, another giant virus distantly related to pandoraviruses, has a spherical virion, also unique among the known viruses ^35^. Strikingly, giant viruses have been identified that encode many more translation system components than the mimivirus, including tupanvirus, which is endowed with a nearly complete translation machinery, minus the ribosome ^36, 37^. These remarkable features notwithstanding, all of the discovered giant viruses retain most of the core NCLDV genes ^16, 36– 39^. These findings further support the monophyly of the NCLDVs, stimulating the formal proposal to classify this group of viruses as the order “Megavirales” ^9, 40^. So far, however, the proposal has not been approved by the International Committee on Taxonomy of Viruses.

In this brief review, we present the results of the phylogenomic analysis of the NCLDVs, including the reconstruction of gene gain and loss events. Giant viruses are found in three strongly supported branches of the NCLDVs and are inferred to have evolved on at least three independent occasions from smaller viruses. For most of the translation-related genes of the giant viruses, phylogenetic analysis shows affinity with different eukaryotic lineages, suggesting piecemeal capture of these genes at different stages of giant virus evolution. Several homologous translation-related genes (for example, aaRSs of the same specificity) appear to have been captured by different giant viruses independently. Thus, evolutionary analysis of the giant viruses provides no support for the fourth domain hypothesis or any other reductive evolution scenario but rather places the emergence of virus gigantism into the general context of the dynamic evolution of the NCLDVs. The evolutionary factors that promote the genomic expansion in multiple lineages of the NCLDVs remain unclear but might have to do with aspects of virus–host interaction in protists that remain to be investigated. One plausible factor that could promote the increase in virion size seems to be adaptation for phagocytosis whereby, to be able to enter the cells of phagocytic protists, such as amoebas, viruses should exceed a minimal particle size, on the order of 1 μM ^41^. Notably, to give further credence to this possibility, it has been shown that marseillevirus virions that are far below the phagocytosis size threshold form “giant” multi-particle aggregates that are phagocytized by amoebas ^42^. Although numerous NCLDVs infect protists that possess cell walls and lack the phagocytic capacity, such as algae ^43^, no giant viruses infecting these organisms have been discovered so far ^44^ and this is compatible with a link between virus gigantism and cell entry by phagocytosis. This entry route could be considered a strategy that allows giant viruses to stay in successful competition with smaller ones that might reproduce faster.

## Reconstruction of the evolution of the NCLDVs and multiple origins of viral gigantism

**Figure 1.**
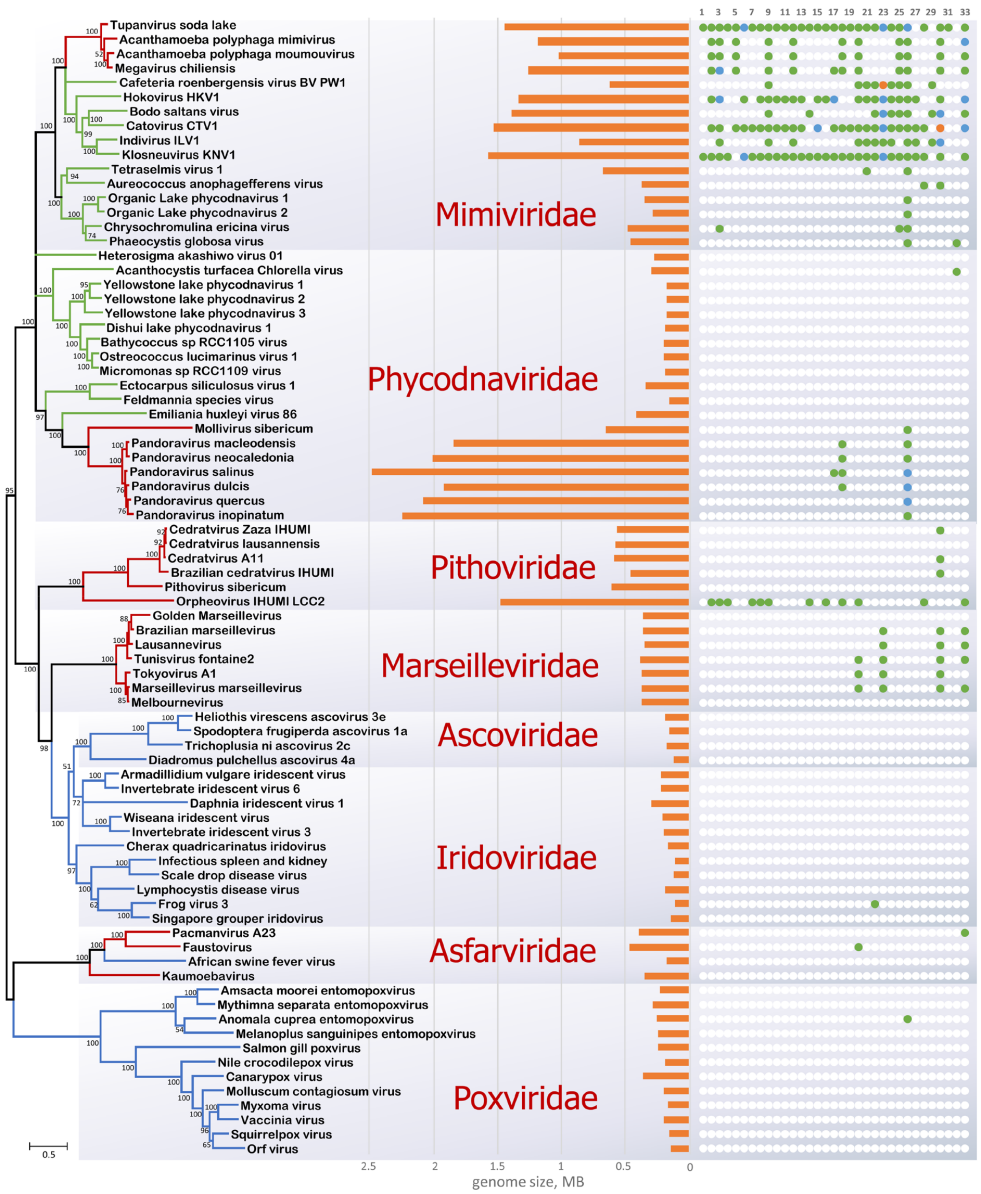
Phylogenetic tree of five genes that are nearly universal in nucleocytoplasmic large DNA virus (NCLDV). The tree was constructed from concatenated multiple alignment of five (nearly) universally conserved NCLDV proteins: DNA polymerase, major capsid protein, packaging ATPase, A18-like helicase, and poxvirus late transcription factor VLTF3. The branch color denotes confirmed or likely hosts: red, Amoebozoa; green, other protists; blue, Metazoa. The tree was constructed by using the FastTree software ^60^ with default parameters. The numbers at the internal branches indicate local likelihood-based support (percentage points); the branches with support below 50% were collapsed. Scale bars represent the number of amino acid (aa) substitutions per site. The middle panel shows genome length, on the scale shown in the bottom of the figure. The right panel shows the distribution of translation-related genes among the NCLDVs: 1–19, aminoacyl tRNA synthetases (1, Ala; 2, Arg; 3, Asn; 4, Asp; 5, Cys; 6, Gln; 7, Gly; 8, His; 9, Ile; 10, Leu; 11, Lys; 12, Met; 13, Pro; 14, Phe; 15, Thr; 16, Ser; 17, Trp; 18, Tyr; 19, Val); 20–33, translation factors (20, eIF-1/SUI1; 21, eIF1a; 22, eIF2a; 23, eIF2b; 24, eIF2g; 25, eIF4a; 26, eIF4e; 27, eIF4g; 28, eIF5a; 29, eIF5b; 30, EF1a; 31, aEF2; 32, eEF3; 33, eRF1). Green, blue, and orange circles represent one, two, or three proteins of the respective family encoded in a genome.

Among the about 40 core genes of the NCLDVs, only three—namely those for the family B DNA polymerase, primase-helicase, and a (predicted) transcription factor—are conserved in all available NCLDV genomes. Thus, there is a limited choice of (nearly) universal markers for constructing an NCLDV phylogeny. To obtain a phylogenetic tree for an updated collection of NCLDVs, we used a concatenated alignment of the three universal genes and two genes that are each missing in a single NCLDV group, those for the major capsid protein and the packaging ATPase. The resulting tree includes three major branches: (1) families *Mimiviridae* and *Phycodnaviridae* together with pandoraviruses; (2) families *Pithoviridae*, *Marseilleviridae*, *Iridoviridae*, and *Ascoviridae*; and (3) families *Poxviridae* and *Asfarviridae*, together with the recently discovered viruses that are related to asfarviruses but infect protists. Technically, this is an unrooted tree. Nevertheless, additional evidence strongly suggests that the root can be placed at branch 3 ( Figure 1). Specifically, the sequences of most of the core proteins in branches 1 and 2 are significantly more similar to each other than to any of those in branch 3, and in phylogenetic trees built for individual core NCLDV genes with cellular homologs as outgroups (such as DNA and RNA polymerases), branch 1 typicall joiuns branch 2, to the exlclusion of branch 3 ^45^. Giant viruses, arbitrarily defined as those with genomes larger than 500 kb, appear in two of the three branches.

Branch 1 includes most of the giant viruses that appear in two disjoint clades, namely extended family *Mimiviridae* and pandoraviruses ( Figure 1). Within branch 1, the monophyly of two recently delineated groups, each combining giant viruses with much smaller ones, is confidently supported. The first of these groups that has been denoted “extended *Mimiviridae*” ^46, 47^ unites the family *Mimiviridae* that consists entirely of giant viruses with a group of viruses with moderate-size genomes, such as the Organic Lake phycodnaviruses and *Phaeocystis globosa* virus (OLPG group) (originally, these viruses were mislabeled phycodnaviruses, apparently because they originate from habitats dominated by algae ^48^). The OLPG clade that consists mostly of viruses with moderate-size genomes in the range of 350 to 400 kb also includes its own “small giant”, the Tetraselmis virus 1, with a 668-kb genome ^49^. The second non-trivial group within branch 1 includes the giant pandoraviruses and *Mollivirus sibericum* which form a clade with coccolithoviruses, as previously reported ^16, 50^. The coccolithoviruses are generally classified within the family *Phycodnaviridae* ^51^ but, in our current tree, fail to show affinity with the rest of the phycodnaviruses.

Branch 2 encompasses the families *Pithoviridae*, *Marseilleviridae*, *Iridoviridae*, and *Ascoviridae*. Of the three main branches in the NCLDV phylogeny, this one includes the widest range of genome sizes, from about 100 kb in the smallest iridoviruses to more than 1.5 Mb in orpheoviruses, a recently discovered member of the *Pithoviridae* ^52^. The family *Pithoviridae* consists entirely of giant viruses.

Branch 3 consists of two distinct clades: asfarviruses (effectively, numerous strains of African swine fever virus) joined by their larger, protist-infecting relatives—faustovirus ^53^, pacmanvirus ^54^, and kaumoebavirus ^55^—and poxviruses. The switch from protists to animal hosts appears to have occurred twice during the evolution of this branch. These switches are likely to have taken place late in asfarviruses (at least, judging from the current knowledge that is limited to a group of closely related viruses that infect a single mammalian species ^56^) and early at the base of the poxvirus clade which consists of numerous viruses infecting both arthropods and vertebrates, two animal phyla that radiated from the common ancestor more than half a billion years ago ^57^. Branch 3 does not include any giant viruses, although the protist-infecting viruses in the Asfar-like clade approach the (arbitrary) gigantism threshold ( Figure 1).

**Figure 2.**
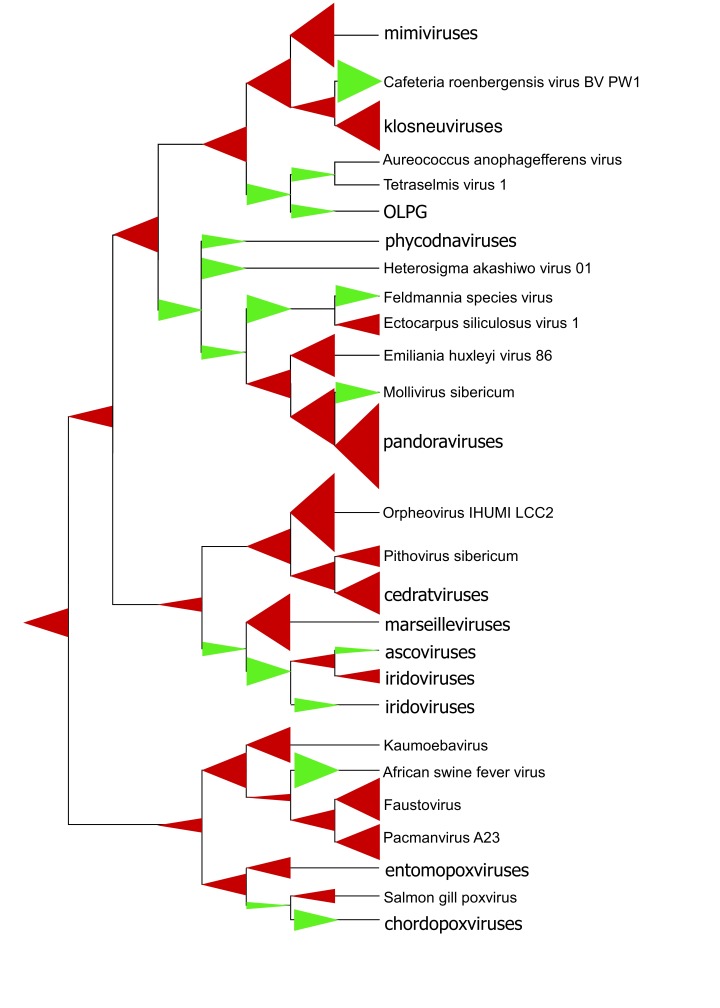
Gene gain and loss in the evolution of the nucleocytoplasmic large DNA virus (NCLDV) and multiple origins of giant viruses. The tree topology is from the phylogeny of five nearly universal genes ( Figure 1). The maximum likelihood reconstruction was produced by using the GLOOME software ^61^, from the mapping of 5284 clusters of homologous NCLDV genes onto the tree leaves (extant viruses). Red triangles show gene gains, and green triangles show gene losses. The size of a triangle is roughly proportional to the maximum likelihood estimate of the number of gains or losses.

Under the assumption of the evolution of all NCLDVs from a common virus ancestor, the phylogeny of the (nearly) universal genes can be used as a scaffold for the reconstruction of gene gain and loss events that occurred in the course of virus evolution along the branches of the tree. In brief, all the genes in the compared genomes (in this case, all complete NCLDV genomes) form families of both (putative) orthologs and singletons are mapped to the leaves of the tree, and maximum likelihood estimates of the numbers of genes gained and lost are obtained for each branch by using a dedicated algorithm ^16, 39, 58, 59^. This reconstruction reveals a striking picture of turbulent evolution of the NCLDVs which is dominated by gene gain, although many branches are associated with substantial gene loss ( Figure 2). Not unexpectedly, the branches that include giant viruses—especially pandoraviruses and orpheovirus—come across as the most prominent gene gainers ( Figure 2). Apparently, the evolution of the three giant virus branches—mimiviruses, pandoraviruses, and pithoviruses—involved gradual build-up of increasingly large and complex virus genomes, independently leading to virus gigantism. Notably, however, this trend appears to have been reversed on at least three occasions: in the OLPG group and in Cafeteria roenbergensis virus (CroV) within the mimivirus branch and in mollivirus within the pandora-coccolithovirus branch ( Figure 2). In the OLPG group, only Tetraselmis virus 1 remains a giant, whereas the rest of the viruses have shrunken to a moderate size. Both CroV and mollivirus remain giants despite apparently losing many genes in the course of evolution from their respective, most recent traceable ancestors. Apart from the branches containing giant viruses, there is a consistent trend of gene loss among those NCLDVs that apparently have switched from protist to animal hosts, namely irido-ascoviruses, asfarviruses, and poxviruses ( Figure 2). Taken together, these findings reveal a highly dynamic evolution of the NCLDVs and strongly suggest that giant viruses evolved from simpler viruses with smaller genomes on many independent occasions ^16^. The opposing model of reductive evolution is disfavored by the maximum likelihood reconstruction method and indeed appears improbable because it would imply enormous ancestral “genomes of Eden” that would include thousands of genes currently represented in individual branches of the NCLDVs, particularly in giant viruses. Other lines of evidence seem to converge on the gene accretion scenario for the origin of giant viruses. In particular, it was recently demonstrated that the number of proteins containing repetitive domains that are characteristic of giant viruses scales almost linearly with the genome size, suggesting that the proliferation of genes encoding repetitive proteins is part and parcel of the evolutionary growth of the virus genomes ^62^.

The conspicuous lack of giant viruses in animals and the apparent genome shrinkage associated with the host range transition from protists to animals in three distinct branches of the NCLDVs appear to hint at some factors that promote virus genome expansion in unicellular eukaryotes. However, the specific aspects of virus–host interactions in protists that could be important for the emergence of giant viruses remain obscure.

## Translation system-related genes in giant viruses: a complex history of acquisitions from eukaryotic hosts

The evolutionary reconstruction discussed in the previous section points to the multiple origins of giant viruses from smaller NCLDVs via the gain of multiple genes. This appears to be a strong indication of genome complexification rather than genome reduction being the dominant process in giant virus evolution. However, these are bulk quantitative analyses of genome evolution. What about the specific subset of genes that have been most important for the fourth domain hypothesis, namely those encoding translation system components?

Normally, with the exception of tRNA genes present in some bacteriophages ^63, 64^, viruses lack translation-related genes. Indeed, the reliance on the translation system of the host is one of the defining features of viruses that have been branded “capsid-encoding” organisms as opposed to cellular life forms, the “ribosome-encoding organisms” ^65^. The giant NCLDVs are the only major exception to this rule currently known ( Figure 1). The discovery of the mimivirus genes encoding aaRSs and some translation factors was an almost shocking surprise that triggered the fourth domain hypothesis ^5, 9– 13, 66^. Actually, the repertoire of the translation system components in the mimiviruses is modest compared with those of the recently discovered klosneuvirus and tupanvirus which encode nearly full sets of the proteins and tRNAs involved in translation, except for the ribosomal RNA and proteins ( Figure 1) ^36, 37^. The dramatic variance of the representation of translation-related genes among the members of the family *Mimiviridae* is particularly conspicuous ( Figure 1). Specifically, tupanvirus, a close relative of the mimiviruses, encodes the complete set of 20 aaRSs, along with 11 translation factors, whereas the mimiviruses proper have seven aaRSs and five translation factors at most ( Figure 1). Similarly, klosneuvirus encodes 19 aaRSs ^37^, whereas the related Bodo sultans virus has only two ^67^. The mimivirus branch sensu lato is by far the richest cache of translation-related genes among the NCLDVs (and actually among all viruses). Outside this branch, the only member of the NCLDVs with numerous translation-related genes is orpheovirus, the member of the family *Pithoviridae* with the largest genomes. Pandoraviruses (despite having the largest genomes among all viruses), the rest of the pithoviruses, and marseilleviruses have a minimal representation of the genes from this functional class, whereas the rest of the NCLDVs have virtually none ( Figure 1). Even by themselves, without a detailed phylogenetic analysis, these striking differences among the NCLDVs suggest that evolution of the translation-related genes in giant viruses involved extensive and repeated gain and loss.

At first glance, the high abundance of translation-related genes in klosneuvirus and tupanvirus would appear to be best compatible with the origin of these giant viruses by reductive evolution from a cellular ancestor, perhaps one from the elusive fourth domain of cellular life. However, phylogenetic analysis of individual translation-related genes clearly paints a different picture ^15, 16^. Three major trends are apparent in the phylogenies of the translation-related genes of the NCLDVs ^16, 37, 44^: (i) contrary to the early phylogenies for the mimivirus aaRS and limited sets of cellular homologs ^5^, in the extended phylogenies, most of these genes cluster with different eukaryotic lineages, deep within the eukaryotic tree, which suggests relatively late acquisition from eukaryotic hosts; a few viral translation-related instead appear to be of bacterial origin; (ii) those translation-related genes that are represented in multiple groups of NCLDVs are polyphyletic, indicating that these genes were acquired repeatedly and independently by different groups of the NCLDVs; (iii) in the translation-related gene phylogenies, the NCLDVs are mixed in different combinations, suggesting multiple intervirus gene exchanges.

**Figure 3.**
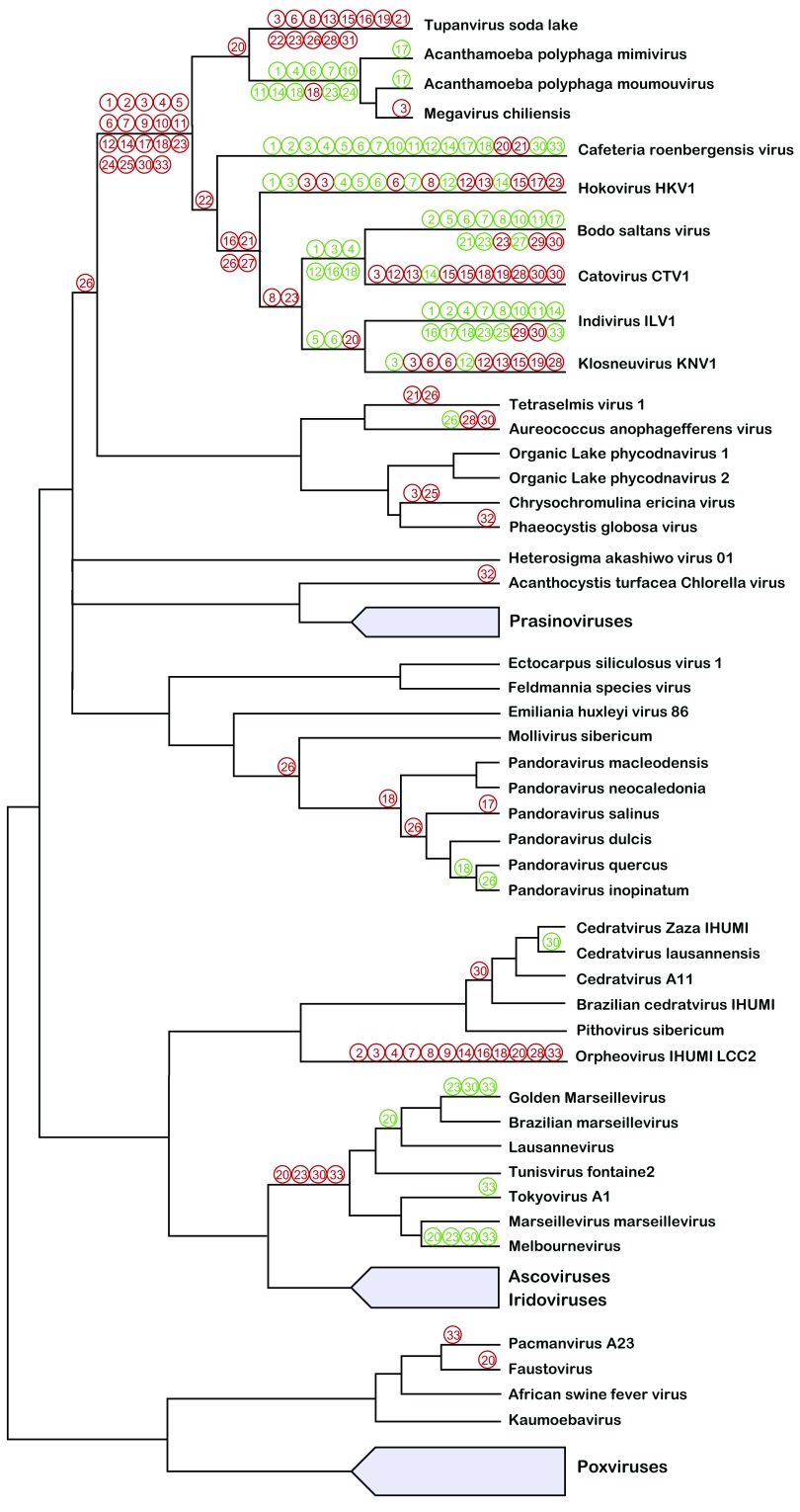
Translation-related gene gain and loss in the evolution of the nucleocytoplasmic large DNA virus (NCLDV). Inferred gains of translation-related genes is shown by red circles, and the loss of translation-related genes is shown by green circles. The inferences are based on previously analyzed phylogenetic trees ^37, 44^. The translation-related genes are numbered as in Figure 1.

Some of the translation-related genes can be inferred to have been captured relatively early in the evolution of the NCLDVs ( Figure 3). For example, eukaryotic translation initiation factor eIF4e apparently was independently acquired by the common ancestors of the mimiviruses and the pandora-mollivirus clade. The phylogeny of tyrosyl-tRNA synthetase (TyrS), one of the most common translation-related genes in NCLDVs, implies that this gene was acquired by giant viruses from different eukaryotes on five distinct occasions ( Figure 3). The primary acquisitions of TyrS can be traced to (i) the common ancestor of mimiviruses and klosneuviruses, (ii) the ancestral pandoravirus, and (iii) the orpheovirus lineage. Additionally, the TyrS gene appears to have been displaced by distinct eukaryotic homologs in the mimiviruses and in catovirus, a member of the klosneuvirus group. A simpler history can be inferred for isoleucyl-tRNA synthetase that apparently was independently captured by orpheovirus and by the common ancestor of the family *Mimiviridae* ( Figure 3). Other translation-related genes similarly show mixed histories of early capture by emerging giant viruses, parallel acquisition by different lineages of the NCLDVs, and occasional losses ( Figure 3).

Taken together, the results of phylogenetic analyses of the translation-related genes represented among the NCLDVs reveal a complex history of parallel gene capture, primarily from eukaryotic sources, that appears incompatible with the origin of these genes from a decaying translation system of a single cellular ancestor via reductive evolution. Instead, it appears to be, above all, a story of multiple, convergent acquisition of translation-related genes from diverse eukaryotes as well as, in a few cases, bacteria and archaea, accompanied by some losses and intervirus gene exchanges. The trend of convergent capture of genes for translation system components implies a selective pressure for the retention of these genes in the giant virus genomes. A striking manifestation of this trend is the accretion of a near complete translation system (without the ribosome) in tupanvirus and klosneuviruses. The biological underpinnings of this apparent evolutionary pressure are obscure. A logical explanation appears to be that the protist hosts respond to the giant virus infection by shutting down their translation system, thus preventing the virus reproduction and possibly leading to cell quiescence or death. Accordingly, to overcome this line of defense, viruses would strongly benefit from being able to replenish the translation system, at least partially restoring translation to produce virus proteins. Experimental validation of this hypothesis requires extensive experimentation on protist models of giant virus reproduction, which is challenging because of the paucity of available genetic and biochemical tools.

## Concluding remarks

The giant viruses are oddities in the virosphere and, at least size-wise, might appear “cell-like”; both their particle and genome sizes are well within the range characteristic of prokaryotes. Yet reconstructions of their genome evolution suggest that these giants of the virus world are its regular denizens with origins traceable to multiple lineages of smaller, simpler viruses. Similarly to other “simply large” viruses, the giant viruses appear to evolve mostly by capture of various genes from the hosts. The difference between the giant viruses and the rest of the virosphere appears to be quantitative rather than qualitative. Furthermore, the apparent evolution of the giant viruses from smaller ones via gene accretion seems to continue a more general and deeper evolutionary process whereby the NCLDVs themselves evolved from much smaller viruses such as eukaryotic polinton-like viruses and ultimately prokaryotic tecti-like viruses ^68, 69^.

Why do giant viruses get so big? Part of the answer is likely to be simply because they can. Apparently, the acquisition of an autonomous, robust replication system by the ancestral NCLDVs unlocked the door for the genome expansion ^69^. Furthermore, protist hosts seem to represent a “melting pot” of gene exchange and acquisition, where the evolving giant viruses can capture genes from the host, the endosymbiotic bacteria it harbors, and other viruses ^70, 71^. We do not know whether there are any hard limits to the extent of virus genome growth. So far, the largest icosahedral viruses—klosneuvirus and orpheovirus—encompass genomes of about 1.5 Mb; larger genomes have been observed only in pandoraviruses with their unusual amphora-shaped virions. It would be premature, though, to conclude that the size limit for icosahedral virions has been reached. There seems to be a good chance that many new giant viruses will be discovered, both with typical and with irregular virion shapes. Furthermore, a major conclusion from the phylogenomic reconstructions is that giant viruses have evolved from simpler ones on many independent occasions. Accordingly, it seems only a matter of time before giant viruses spring up in lineages where they have not been detected so far, such as phycodnaviruses, marseilleviruses, and Asfar-like viruses. A more difficult question is whether giant viruses might exist in multicellular organisms, in particular in animals. As pointed out above, all currently known groups of NCLDV-infecting animals appear to have undergone some degree of genome contraction during their evolution from ancestral protist viruses. It cannot be ruled out that, in animals, the pressure for virus genome compactification is stronger than in protists; if so, it will be interesting to understand the nature of such pressure.

In principle at least, the evolution of giant viruses seems to resemble that of “giant” bacteria, those with genomes larger than 10 Mb. The giant bacteria independently originated in multiple phyla, such as cyanobacteria, actinomycetes, and deltaproteobacteria, and generally are microbes that inhabit complex, changing environments and go through complex life cycles ^72^. There might be a clue here to the conditions that promote the emergence of giant virus genomes as well, although currently there are no clear ideas on the nature of environmental and developmental complexity that might underlie the genome expansion among the NCLDVs.

The study of the NCLDV evolution does not seem to provide any support for iconoclastic ideas on reductive evolution of giant viruses from cells (be it a fourth domain of life or not), in agreement with the conclusions already reached by early phylogenetic analyses of the mimivirus genes ^14, 19, 21^. The virus–cell separation seems to remain the fundamental divide between autonomous and parasitic biological agents ^73, 74^. Nonetheless, there is no doubt that the study of giant viruses has the potential to uncover plenty of fascinating biology.
